# Exploring Actinobacteria Associated With Rhizosphere and Endosphere of the Native Alpine Medicinal Plant *Leontopodium nivale* Subspecies *alpinum*

**DOI:** 10.3389/fmicb.2019.02531

**Published:** 2019-11-08

**Authors:** Martina Oberhofer, Jaqueline Hess, Marlene Leutgeb, Florian Gössnitzer, Thomas Rattei, Christoph Wawrosch, Sergey B. Zotchev

**Affiliations:** ^1^Pharmaceutical Biotechnology, Department of Pharmacognosy, University of Vienna, Vienna, Austria; ^2^Division of Systematic and Evolutionary Botany, Department of Botany and Biodiversity Research, University of Vienna, Vienna, Austria; ^3^Department of Microbiology and Ecosystem Science, University of Vienna, Vienna, Austria

**Keywords:** endophytes, rhizosphere, Edelweiss, Actinobacteria, alpine medicinal plant

## Abstract

The rhizosphere of plants is enriched in nutrients facilitating growth of microorganisms, some of which are recruited as endophytes. Endophytes, especially Actinobacteria, are known to produce a plethora of bioactive compounds. We hypothesized that *Leontopodium nivale* subsp. *alpinum* (Edelweiss), a rare alpine medicinal plant, may serve as yet untapped source for uncommon Actinobacteria associated with this plant. Rhizosphere soil of native Alpine plants was used, after physical and chemical pre-treatments, for isolating Actinobacteria. Isolates were selected based on morphology and identified by 16S rRNA gene-based barcoding. Resulting 77 Actinobacteria isolates represented the genera *Actinokineospora*, *Kitasatospora*, *Asanoa*, *Microbacterium*, *Micromonospora*, *Micrococcus*, *Mycobacterium, Nocardia*, and *Streptomyces*. In parallel, Edelweiss plants from the same location were surface-sterilized, separated into leaves, roots, rhizomes, and inflorescence and pooled within tissues before genomic DNA extraction. Metagenomic 16S rRNA gene amplicons confirmed large numbers of actinobacterial operational taxonomic units (OTUs) descending in diversity from roots to rhizomes, leaves and inflorescences. These metagenomic data, when queried with isolate sequences, revealed an overlap between the two datasets, suggesting recruitment of soil bacteria by the plant. Moreover, this study uncovered a profound diversity of uncultured Actinobacteria from Rubrobacteridae, Thermoleophilales, Acidimicrobiales and unclassified Actinobacteria specifically in belowground tissues, which may be exploited by a targeted isolation approach in the future.

## Introduction

Microbial habitats in plant and in soil encompass the bulk soil, the rhizosphere, and the endosphere (Compant et al., 2010; Wang M. et al., 2016). The bulk soil is a meso- to oligotrophic habitat characterized by pronounced heterogeneity in physical properties, nutrient availability, and other abiotic factors (Lemanceau et al., 2017) and therefore, may both enhance microbial diversity, and support proliferation of particular species (Gottel et al., 2011; Wang M. et al., 2016). In contrast, the rhizosphere is in immediate contact with the plant roots and is actively enriched by a complex mixture of carbon/nutrient sources, such as amino acids, sugars, and other nutrients provided by the plant, in a process known as rhizodeposition (Hütsch et al., 2002; Bais et al., 2006). This environment attracts a plant species-specific microbial community (Minz et al., 2013; Essarioui et al., 2017) and is then modified by both the plant and microorganisms. The assemblies of microorganisms present in the rhizosphere are dependent on the soil type, the host species, the host plant genotype, and root system architecture (Ofek-Lalzar et al., 2014; Pérez-Jaramillo et al., 2017; Saleem et al., 2018). The endosphere is adjacent to the rhizosphere and is intimately linked to it by acquiring its microbial inhabitants, the endophytes, not exclusively, but to great extent through horizontal transmission (Frank et al., 2017). Some root endophytes may also result from vertical transmission through seeds and subsequently migrate into root tissues (Johnston-Monje and Raizada, 2011; Mitter et al., 2017). In contrast to pathogens, endophytes live in plant tissues without causing harm to their host. Particularly bacterial endophytes often originate from the rhizosphere of their host plant (Compant et al., 2010; Hardoim et al., 2015). The endosphere entails the entire microbial community that inhabits the interior of the host plant. Generally, host plants provide an environment with high nutrient content to their microbial inhabitants and shelter them from adverse environmental factors (Compant et al., 2010; Wang M. et al., 2016). Thus, endophytes experience high selective pressure competing for their beneficial, but spatially limited habitat (Caraballo-Rodríguez et al., 2017). Within the endosphere, different plant tissues are characterized by their specific chemoprofiles and tissue architecture, which can act as “filters” and attract different subsets of the endophyte community. Both the rhizosphere and the endosphere impose strong selective forces on microbes, which promote highly interactive communities. This suggests involvement of secondary metabolites in interactive signaling affecting antibiosis (Yim et al., 2007), quorum sensing and biofilm formation (Abisado et al., 2018). Hence, these two symbiotic habitats bear prime potential for the discovery of novel natural products (Strobel et al., 2004; Tiwari and Gupta, 2013).

*Leontopodium nivale* subsp. *alpinum* (Cass.) Greuter, syn. is commonly known as Edelweiss, a hemicryptophytic herbaceous plant species of family Asteraceae. The focal area of diversity in the genus *Leontopodium* is the Sino-Himalayan region, where several species are used as medicinal plants (Safer et al., 2011). Edelweiss occurs in subalpine to alpine grassland communities (Hörandl et al., 2011) and is currently believed to be monotypic for the genus in Europe. The plant has been used historically as a medicinal herb against numerous ailments in traditional medicine. It was used to treat dysenteria in humans and animals, bronchitis, diarrhea, fever, and was generally used for its anti-inflammatory and antibacterial properties (Dobner et al., 2003). Recent studies show that the root extract exhibits analgesic effects in the rat paw edema test (Speroni et al., 2006). Leontopodic acid, one of the active compounds of the plant, is a potent antioxidant (Schwaiger et al., 2005). Moreover, leoligin is a plant lignan compound that has been shown to inhibit intimal hyperplasia in venous bypass grafts by reverse cholesterol transport without showing signs of cytotoxicity (Reisinger et al., 2009; Wang L. et al., 2016). However, because of overharvesting, Edelweiss became threatened and subsequently its protection status prevents its collection and use as herbal remedy in Europe. In a yet increasing number of medicinal plants, endophytes are being discovered producing either precursor molecules or the actual bioactive compounds previously attributed to their host plant (Köberl et al., 2013; Golinska et al., 2015), which may unlock possibilities for their biotechnological production and relief the focus on the threatened plant.

Recently, medicinal plants have come to the forefront of bioprospecting in search for new or unusual sources for rare Actinobacteria (Golinska et al., 2015), whereby the term rare in this context specifies Actinobacteria phylogenetically distant to *Streptomyces* and that are not commonly isolated (all genera except *Streptomyces*) (Kurtböke, 2003; Janso and Carter, 2010; Khanna et al., 2011; Jose and Jebakumar, 2013). Actinobacteria are Gram-positive bacteria, which have a characteristically high GC-content in their DNA and are ubiquitous in nature. Their habitats range from diverse terrestrial to aquatic ecosystems, but they are also often found associated with other organisms and live in extremophile environments (Quintana et al., 2013). Actinobacteria are recognized as highly prolific producers of secondary metabolites. Bioactive molecules produced by Actinobacteria include antitumor, antiviral, antiparasitic, insecticidal, antibacterial, and antifungal and immunomodulatory agents and herbicides (Barka et al., 2016). Most notably, they are the origin of the majority of all antibiotic compounds currently known (Baltz, 2008).

Numerous studies on Actinobacteria in medicinal plants were conducted in Asia, where the use of herbal remedies is deeply anchored in culture and medicine since millennia, e.g., Ayurveda, Traditional Chinese Medicine (Qin et al., 2009; Zhao et al., 2012). Few studies are available on medicinal plants native to South America, Australia, and the Paleotropics (Golinska et al., 2015; Caraballo-Rodríguez et al., 2017), however, investigations on temperate medicinal herbs are yet underrepresented. Among these, studies on alpine medicinal plants are especially rare. Hence, we hypothesized that *L. nivale* subsp. *alpinum* and its rhizosphere may serve as untapped habitats for rare Actinobacteria that could be of major interest for bioprospecting and drug discovery.

In this study, Actinobacteria associated with three Edelweiss plants were explored with a dual concept consisting of targeted isolation from rhizosphere soil suspension and a culture-independent approach exploring the endophytic communities of different plant tissues. Phylogenetic analysis revealed an overlap between the rhizosphere isolates and plant endophytic Actinobacteria identified using 16S rRNA gene amplicon sequencing. Endophytic actinobacterial community of Edelweiss appears to be rich in rare and probably underexplored species, which can be targeted for isolation in the future bioprospecting efforts aimed at drug discovery.

## Materials and Methods

### Sample Collection and Treatment

Special permission for collecting *L. nivale* subsp. *alpinum* and rhizosphere soil samples was issued by the Environmental Agency Austria in 2015. Three plant individuals and their adjacent rhizosphere soils were collected at the Mount Rax in the Austrian Alps on September 26, 2015 at 1624 m above sea level close to Praterstern at the coordinates N474258.34 and E154542.63. To avoid accidentally harvesting clones originating from laterally arising leaf rosettes on rhizomes of the same plant individual, plants were chosen to be a minimum of 20 m apart from each other. Plants and soils were transported under cooled conditions and upon arrival stored at 4°C until processing. Plants were dissected in leaves, inflorescences, rhizomes, and roots and pooled within tissues. Plant tissues were first carefully rinsed with tap water and then surface sterilized using an aqueous solution of sodium hypochlorite (2% active chlorine) for 10 min. Tissues were then washed twice with autoclaved distilled water and treated with 70% ethanol for 1 min and rinsed with sterile water. Surface sterilized plant tissues were ground in a mortar and 20% glycerol added before storing at −80°C until further processing.

### Soil Suspensions and Pre-treatments

Soil stock suspensions were produced using 250 mg air dried rhizosphere soil and 2.25 ml autoclaved distilled water for each treatment. Pre-treatments were applied to diminish fast growing and abundant soil bacteria that would hinder slow growing Actinobacteria isolation by overgrowth (Kurtböke, 2003). Dry heat pre-treatment was achieved with dry rhizosphere soil, which was exposed to 100°C for 1 h. Then, soil was re-suspended in 2.25 ml of sterile water after cooling to room temperature. The mixture was vortexed and immediately used for serial dilutions. The phenol pre-treatment was accomplished by adding 4.5 ml of a 5 mM phosphate buffer (pH 7) containing 1.5% phenol to one of the four soil stock suspensions described above. This mixture was kept at 30°C in a water bath for 30 min and was then used for producing serial dilutions. Inoculated plates from this pre-treatment were stored in the fume hood covered with aluminum foil at room temperature for 3 days to allow the safe evaporation of the phenol. Microwave pre-treatment was performed by exposing the soil stock suspension to microwaves for 30 sec (2460 MHz frequency, 100 W power). No pre-treatment as a control was achieved by untreated soil stock suspension as origin of further dilutions. For all isolation plates and dilutions applied that 100 μl of pre-treated soil suspension was spread on the nutrient agar surface. All nutrient media plates were incubated at 28°C for microbe isolation, phenol treated plates joined at day four.

### Isolation Media

Selective nutrient media were employed as means of targeting rare Actinobacteria genera. Media presented specific carbon (C) and nitrogen (N) sources, micronutrients and in some media addition of B-vitamins (Table 1; Kurtböke, 2003; Magnúsdóttir et al., 2015). Two media (MGA, LSV) required the addition of soil extract (Table 1), which was prepared from common garden soil at the medicinal plant garden of the Department of Pharmacognosy at the University of Vienna. Soil was dried in a beaker at 120°C for 90 min and ground with mortar and pestle. 400 g of ground soil was used following the protocol for soil extract medium without the addition of agar^1^. Resulting extract was stored at −20°C until use.

**TABLE 1:** Carbon and nitrogen sources, B-vitamins, and soil extract in selective media targeting Actinobacteria (Kurtböke, 2003).

**Media**	**C-source**	**N-source**	**B-vitamins**^1^
AV	Glucose	Arginine	+
	Glycerol		
MC	Glucose	NaNO_3_	
MGA-SE^2^	Glucose	L-Asperagine	
	Soil extract		
GAC	Glucose	L-Asperagine	
HV	Humic acid	Humic acid	+
LSV-SE^2^	Lignin	Soy bean flour	+
	Soil extract		

*^1^Composed of 0.5 mg each of thiamine-HCl, riboflavin, niacin, pyridoxin-HCl, inositol, Ca-pantothenate, p-aminobenzoic acid and 0.25 mg of biotin. ^2^SE = soil extract.*

### Antibiotic Treatment

Media were supplemented with either no antibiotics, a dual combination of nystatin and cycloheximide (NC) or a triple combination of nystatin, cycloheximide, and novobiocine (NCN). Nystatin (20 μg/ml) and cycloheximide (20 μg/ml) suppress fungi, which can rapidly colonize entire isolation plates. Novobiocine (100 μg/ml) acts against certain Gram-negative and most Gram-positive bacteria targeting abundant unicellular bacteria in soil suspensions.

### Experimental Design

The experiment included a total of 864 isolation plates resulting from factorially combining four pre-treatments of soil suspension, six serial dilutions thereof, six media selective for rare Actinobacteria and three antibiotic treatments. Each combination was replicated twice. After inoculation, plates were investigated twice per week manually and isolates matching morphological criteria of Actinobacteria were picked selectively with tooth picks and streaked out to new plates with the same media and antibiotic regime as present on the original isolation plates. Purified strains were then inoculated onto solid media facilitating spore formation. Spores were harvested from each sporulating strain and re-suspended in 20% glycerol and stored at −80°C (Kieser et al., 2000). Actinobacteria with no spore formation were maintained as scrapings as described above. Simultaneously, liquid cultures were inoculated with the strains using Tryptic Soy Broth (TSB) media as default and 2x Yeast Extract/Tryptone (2xYT) media, in case no sufficient growth was observed in TSB. Cell suspensions were centrifuged and the pellets stored at −20°C until genomic DNA extraction.

### Sanger Sequencing of Isolates and Phylogenetic Analyses

**FIGURE 1:**
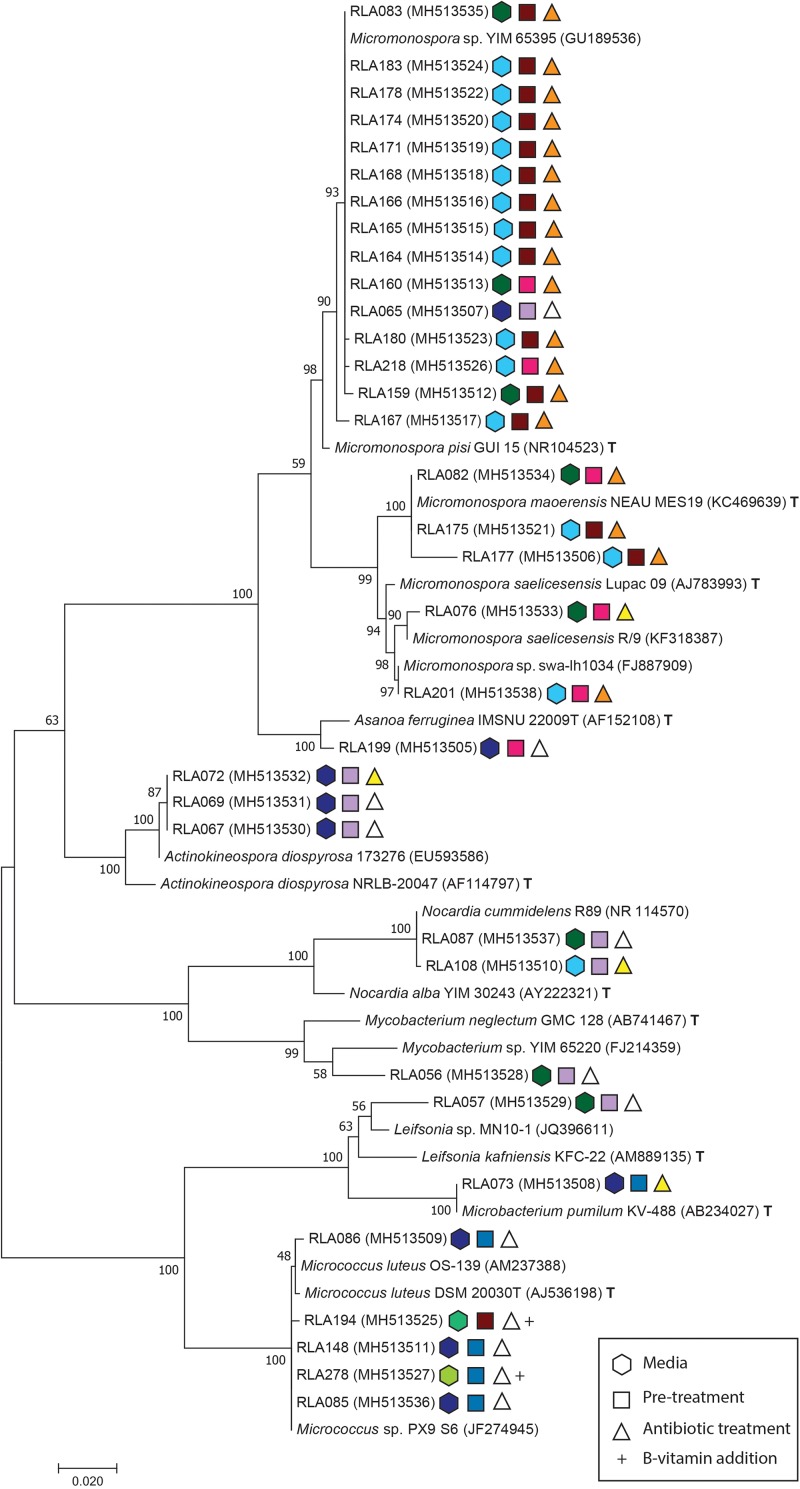
Maximum composite likelihood analysis (MCL) of Actinobacteria isolates from the rhizosphere of *Leontopodium nivale* subsp. *alpinum* based on Tamura and Nei (1993) model with Gamma distribution and invariate sites using Mega software version 7.0.18 (Kumar et al., 2016). The phylogenetic tree contains experimental sequences (RLA) with their GenBank accession numbers, their closest matches in GenBank Blast search and, respectively, closest type strains (T). The tree is drawn to scale with branch length representing the substitutions per site. Bootstrap support values were calculated with 1000 replicates. Isolates identify by the prefix -RLA followed by an individual isolate number and GenBank accession numbers in brackets. Conditions for each isolation are depicted as symbols behind accessions. Hexagonal symbols encode selective media (light blue = MGA-SE; dark blue = MC; turquois = AV; dark green = GAC; light green = LSV-SE), square symbols describe pre-treatments (dark red = microwave; blue = dry heat; pink = phenol; lilac = control) and triangles symbolize antibiotic treatments (orange = NCN; yellow = NC; white = control). B-vitamin addition as specified in Table 1 is indicated by a + behind the symbols of isolates.

**FIGURE 2:**
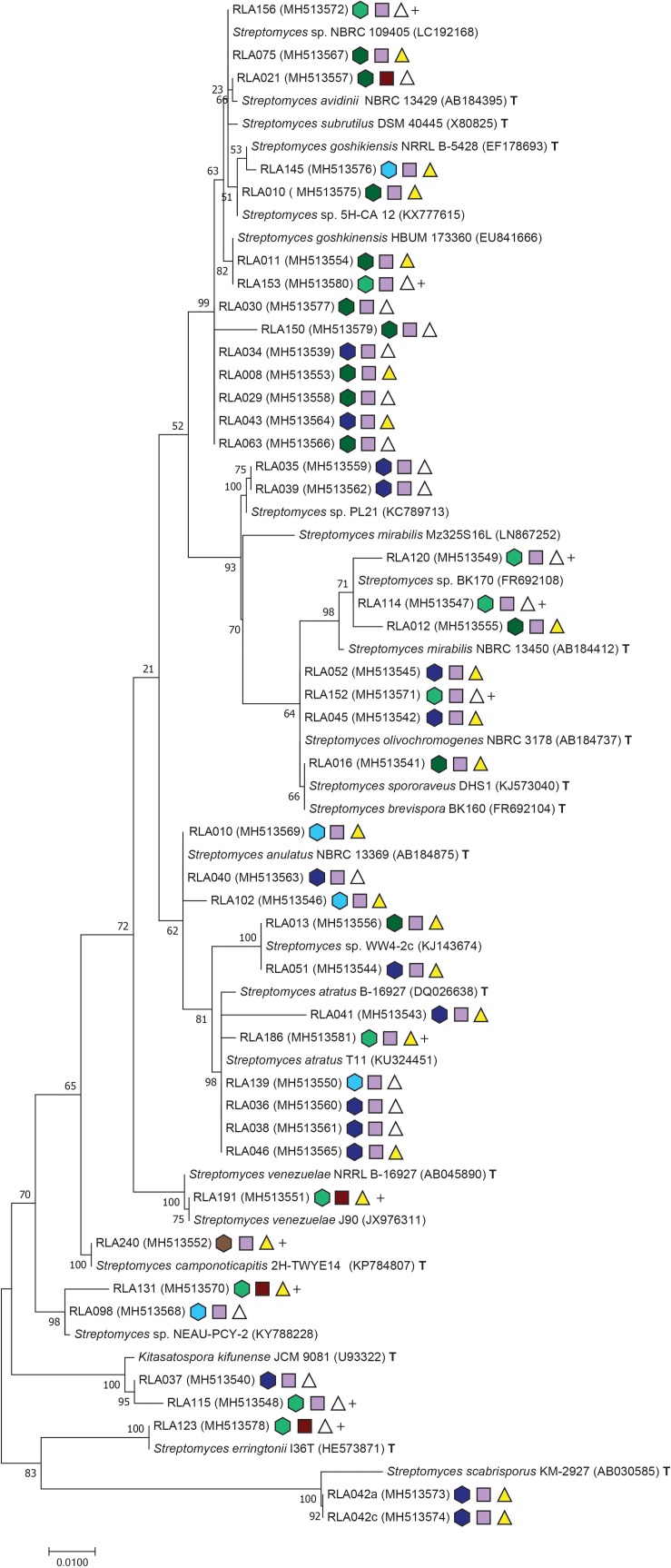
Maximum composite likelihood analysis clustering of *Streptomyces* isolates from Edelweiss rhizosphere. Phylogentic tree reconstruction was performed with Tamura (1992) as best fitting evolutionary model with invariant sites and Gamma distribution using Mega software version 7.0.18 (Kumar et al., 2016). Further specifications of the phylogeny are congruent to Figure 1.

Pellets of purified microbial strains were extracted using the Wizard SV Genomic DNA purification system following the producer’s instructions (Promega Corporation, Madison, WI, United States). Genomic DNA quality and concentration was evaluated by gel electrophoresis in a 0.8% agarose gel supplemented with GelRed (Biotium, Inc., Fremont, CA, United States). Polymerase chain reaction (PCR) of the 16S rDNA barcoding region for bacteria was performed in a final volume of 40 μl using the standard primers 27F and 1492R (Hongoh et al., 2003) each 1 μl, 0.5 μl Taq polymerase and 4 μl of commercial 10xTaq reaction buffer, 0.5 μl dNTPs and 5% DMSO. Genomic DNA was added depending on concentrations as inferred from gel electrophoresis and was modified accordingly with ddH_2_O to reach the final volume. PCR products were verified with gel electrophoresis and products of expected length and specificity were purified with DNA Clean & Concentrator^TM^-5 (Zymo Research, Irvine, CA, United States) and submitted to sequencing by Eurofins Scientific (Brussels, Belgium). Sequences were then assembled and manually edited and multiple alignments were performed in MEGA version 7.0.18 using ClustalW algorithms (Kumar et al., 2016). Most likely evolutionary models of base exchange were estimated using the same software. Phylogenetic analyses were achieved with maximum composite likelihood analysis (MCL) heuristic method of Neighbor-Join and BioNJ algorithms and were tested for robustness using 1000 bootstrap replicates. Gaps were treated as missing values (Figures 1, 2).

Isolates from the rhizosphere of *L. nivale* subsp. *alpinum* are abbreviated with the prefix RLA- followed by an individual isolate number and GenBank accession numbers in brackets. Isolates were supplemented with their closest matches in GenBank BLASTN2.8.1 (Zhang et al., 2000; Morgulis et al., 2008) and their closest related type strains (T)^2^. Single strand sequences and amplicons shorter than 850 bp were excluded from phylogenetic analyses. For better resolution, the alignment of *Streptomyces* (Figure 2) was analyzed separately from other Actinobacteria genera (Figure 1).

The Actinobacteria alignment comprised 794 informative sites from a total of 53 sequences including 36 isolate strains. The best fitting nucleotide substitution model was TN93 plus invariate sites and Gamma distribution with – lnL = −3535 (Tamura and Nei, 1993). The *Streptomyces* alignment included 1124 informative sites from 42 isolate strains. The total alignment consisted of 67 sequences and Mega7 Modeltest identified T92 with invariate sites and Gamma distribution as best fitting evolutionary model with a likelihood value of – lnL = −3444,35 (Tamura, 1992). All sequences were checked for possible chimera formation twice at Genbank Submission using chimera.slayer within Mothur version 1.35.1 (Schloss et al., 2009) and Uchime (Haas et al., 2011). GenBank accession numbers are listed in Figures 1, 2 (SUB4176280). Phylogenies were made available at Dryad^3^.

### Illumina HiSeq2500 Sequencing

Plant samples were separated into leaves, roots, rhizomes and inflorescences at harvest and were pooled among tissue from three plant individuals. Total genomic DNA was extracted using the FastDNA^TM^ SPIN Kit for Soil DNA Extraction (MP Biomedicals, Santa Ana, CA, United States) according to manufacturer‘s recommendations. Plant tissues were thawed and rinsed once with sterile water to remove excess glycerol. Resulting material was lyophilized for 48 h and 100 mg were weighed in for DNA extraction. Genomic DNA quantity and quality was estimated by gel electrophoresis and Nanodrop lite (Thermo Fisher Scientific, Waltham, MA, United States), adjusted to company’s requirements (>1 mg DNA per sample, >25 ng/ml) and submitted for Illumina HiSeq 2500 Sequencing to CD Genomics (Shirley, New York, NY, United States). Amplicons were acquired with the primers 357F and 806R and entailed the V3 and V4 region of 16S rDNA as paired end reads of 250 bases. This yielded a total of 30,000–50,000 sequences per tissue. Sequences were processed according to the Mothur MiSeq SOP by using mothur 1.39.5 (Schloss et al., 2009; Kozich et al., 2013). Barcodes, primers, adapters, and chloroplast sequences were removed and sequences assembled, whereby all sequences unlike a final length of 380–430 bp were excluded from downstream analyses. Chimeras were identified and removed using chimera.vsearch (Rognes et al., 2016). Resulting sequences were clustered into operational taxonomic units (OTUs) with an identity cutoff of 97%. Taxonomy was assigned using the RDB classifier (Wang et al., 2007) and Silva databases^4^ with a bootstrap cutoff of 80% (Supplementary Figure 1). To mitigate the impact of potential sequencing artifacts, we removed singleton OTUs from further analyses. Original metagenomic sequence data have been deposited at the European Nucleotide Archive [PRJEB32100 (ERP114738)]. The full analysis script is available on GitHub^5^. The trimmed sequence alignment and the phylogenetic analysis of Supplementary Figure 1 is available at Dryad^3^.

### Relating Rhizosphere Isolates to the Endophyte Microbiota Composition

Sanger sequences obtained from each isolate were aligned to Actinobacteria OTUs using PAGAN v.0.61 to analyze rhizosphere isolates together with the endophyte microbiota composition (Löytynoja et al., 2012). Poorly aligning regions were removed from the alignments using trimAl 1.2 (Capella-Gutiérrez et al., 2009) with the -automated1 setting. RAxML v.8.2.10 (Stamatakis, 2014) was used to infer a Maximum Likelihood phylogenetic tree using the option for rapid bootstrap and search for best-scoring ML tree (-f a) with the GTRGAMMA model of evolution. The optimal number of bootstraps was determined using the extended majority rule consensus criterion (-autoMRE) and the search converged after 650 bootstrap replicates.

## Results

### Isolates From Rhizosphere Cultures

Rhizosphere isolates were obtained by applying three pre-treatments (microwave, dry heat, phenol) and no pre-treatment, two different antibiotic combinations (NCN, NC) and no antibiotics and media selective for Actinobacteria combined with serial dilutions. Serial dilutions of soil suspensions were performed from 10^–1^ to 10^–6^. While the absolute numbers of chosen isolates were randomly distributed, the percentage of rare Actinobacteria was highest in the lowest dilution. Besides Actinobacteria, 184 other bacterial and two fungal strains were isolated, but not further investigated.

Phylogenetic analyses included a total of 77 strains isolated from *L. nivale* subsp. *alpinum* rhizosphere soil that were identified by 16S rDNA barcoding as either belonging to rare Actinobacteria or the genus *Streptomyces*. Both groups were separately analyzed to retain proper resolution of branches. MCL analysis was used to infer phylogenies for 34 Actinobacteria isolates (Figure 1) and for 43 *Streptomyces* isolates including the closely related genus *Kitasatospora* (Figure 2). The maximum likelihood analysis of 16S rDNA sequences of Actinobacteria isolates (Figure 1) inferred eight distinct clades and two subclades in agreement with genera delimitations of *Micromonospora* (20 isolates), *Asanoa* (1), *Actinokineospora* (3), *Mycobacterium* (1), *Nocardia* (2), *Leifsonia* (1), *Microbacterium* (1), and *Micrococcus* (5). Modes of isolation are specified by color-coded geometric figures in Figures 1, 2.

The *Streptomyces* phylogeny comprised 16 clades, and one representing the rare actinobacterial genus *Kitasatospora* (Figure 2). Clades represent isolates grouping to *Streptomyces subrutilus* (2 isolates), *Streptomyces avidinii* (1), *S. chinensis* (4), a clade not grouping with any type strain closely (2), *S. mirabilis* (3), and *S. olivochromogenes* (3). One isolate grouped closely to the type strains of *S. brevispora* and *S. spororaveus*, whose 16S rDNA differ marginally from each other. Moreover, another clade formed with *S. anulatus* (3). Three isolates of the next clade were closest related to *Streptomyces* sp. WW4-2c, but lacked any closely related type strain. Furthermore, another clade grouped to *S. atratus* (6), *S. venezuelae* (1), *S. camponoticapitis* (1), *Streptomyces* sp. NEAU-PCY-2 (2, type strain unknown). The rare genus *Kitasatospora* is typically embedded within the *Streptomyces* phylogeny (Zhang et al., 1997) and housed two isolates. One more clade grouped with *S. erringtonii* (1). The clade most divergent to all others is clade 17, whose isolates RLA042a and -042c are related to the plant pathogen *S. scabrisporus*.

### Metabarcoding of Plant Tissues

**FIGURE 3:**
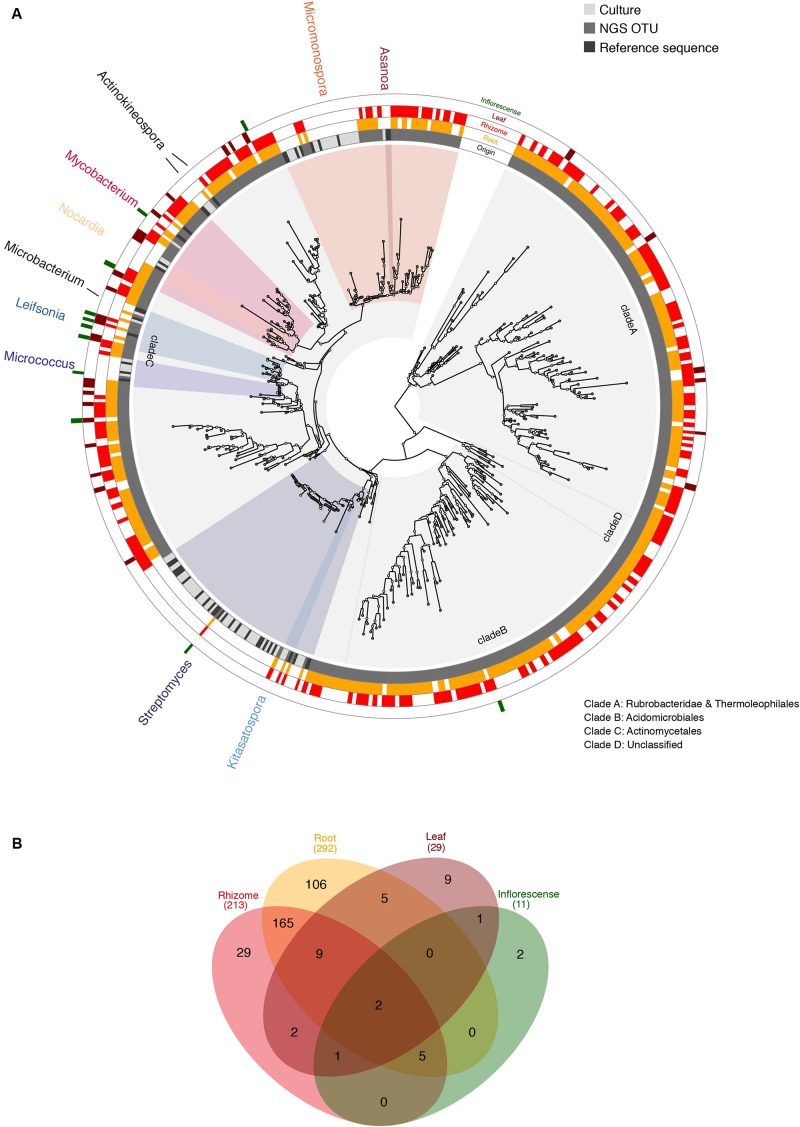
**(A)** ML analysis of combined sequences from Actinobacterial rhizosphere isolates and metagenomic data. The phylogenetic tree includes 16S rDNA sequences derived from rhizosphere isolates (culture, light gray), their closest type strains as references (black) and metabarcoding OTUs (dark gray). Cultured genera are named in the outer ring and highlighted by color coding, based on the last common ancestor spanning all reference sequences assigned to each genus. All indicated genera are supported by bootstrap values >70, except *Streptomyces*. The outermost four concentric rings indicate presence of each OTU according to tissue: root (yellow), rhizome (red), leaf (burgundy), inflorescence (green). This graphic was produced using GraPhlAn (Asnicar et al., 2015). **(B)** Total numbers of OTUs according to tissue with color-coding as in panel **(A)**.

Tissue specific Actinobacteria endophyte communities were investigated with metabarcoding. Based on data from V3 and V4 regions of 16S rDNA, we were able to identify a total of 336 unique Actinobacterial OTUs across the four tissues examined. Of these, 292 were found in the root, 213 in the rhizome, 29 in the leaves, and 11 in the inflorescence (Figures 3A,B). Among these OTUs we were able to identify 127 to the genus level, which yielded a representation of 45 different genera, of which five were shared with the culture isolates, namely *Actinokineospora*, *Nocardia*, *Mycobaterium*, *Micrococcus*, and *Streptomyces*. We also identified 6 OTUs in the Microbacteriaceae and 24 OTUs in the Micromonosporacea that the RDB classifier was unable to resolve to the genus level, suggesting that incomplete recovery of genera also discovered in the culture-based approach (5 of the 10 genera) is at least partially due to the inability to accurately classify to genus level based on short sequencing reads.

In order to infer taxonomic representation among culture isolates and NGS samples independent of automatically assigned classifications, we built a phylogenetic tree including both, cultured isolates and one sequence representing each Actinobacteria OTU (Figure 3A). Phylogenetic analysis revealed four deep clades harboring Rubrobacteridae and Thermoleophilales (clade A; bootstrap 100), Acidimicrobiales (clade B; bootstrap 65), Actinomycetales (clade C; bootstrap 79), and clade D, which houses unclassified Actinobacteria (bootstrap 99). All culture isolates were found to belong to clade C suggesting either a pervasive culture bias in favor of Actinomycetales or enrichment of clades A and B in endophytic tissues compared to the rhizosphere. Clade C also houses 39 of the 45 identified genera, while the remaining 6 are found in clades A and B (3 genera in each). Based on phylogenetic relationships, we found NGS OTUs branching with all culture isolates suggesting near complete recovery of the taxonomic diversity represented among the cultures using NGS data, despite the inability to accurately classify OTUs to genus level in all cases (Figure 3A). Nevertheless, we found a relative overrepresentation of culture-based isolates compared to NGS OTUs, especially in the *Streptomyces* and *Micromonospora* clades (Figure 3A). This is likely due to over-clustering of closely related species in these genera into the same OTU based on strong sequence conservation among isolates in the V3–V4 region sequenced using NGS technology.

**FIGURE 4:**
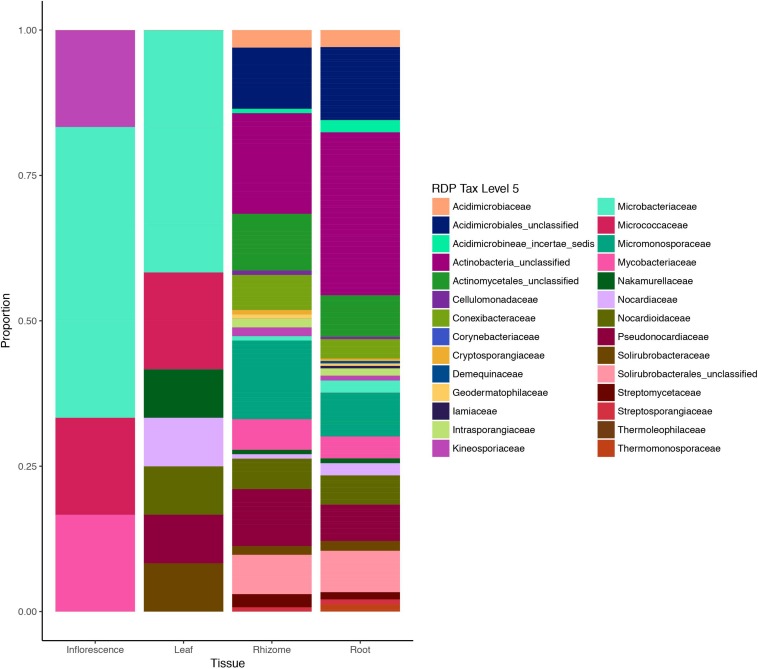
Actinobacteria diversity in different plant tissues of Edelweiss based on metabarcoding data. Tissues of the host plant *Leontopodium nivale* subsp. *alpinum* are illustrated on the *x*-axis (inflorescence, leaf, rhizome, and root) and sequence data are based on presence/absence coding for each OTU. Taxonomic assignments were performed with RDB classifier and Silva database (Wang et al., 2007) and reported at the taxonomic level of families.

We also investigated abundance of different taxa across different tissues based on presence/absence variation of OTUs (Figure 4). Family-level relative abundances showed significant differences between aboveground and belowground tissues (*P* = 8.5e-06; two-sided Fisher exact test with simulated *P*-values based on 1e+07 replicates). Residual plots revealed that this was primarily driven by a dominance of Microbacteriaceae and Micrococcaceae among the leaf and inflorescence tissues, while taxonomic distribution was more balanced and diverse in belowground tissues (Supplementary Figure 2). Differences in taxonomic distribution among belowground tissues were non-significant.

## Discussion

The endosphere of a plant often receives a significant part of its bacterial inhabitants from the rhizosphere through horizontal transmission (Compant et al., 2010; Wang M. et al., 2016). To our knowledge, this is the first study providing initial insights in the presence of rare Actinobacteria from both habitats associated with the ancient and threatened alpine medicinal plant, *L. nivale* subsp. *alpinum*, which have been obtained with a combinatory approach of selective isolation and tissue-specific metagenomic barcoding. Medicinal plants are a promising source of rare Actinobacteria for bioprospecting and have been recently reviewed (Köberl et al., 2013; Golinska et al., 2015), yet temperate and especially alpine plant species remain understudied in this respect.

A range of known selective techniques were employed aiming to target isolation of rare Actinobacteria from the rhizosphere of *L. nivale* subsp. *alpinum*. Pre-treatments of soil such as dry heat, microwave irradiation, and phenol reduce the numbers of abundant co-occurring bacteria (Hayakawa, 2003). Nutrient poor media with specific C- and N-sources (Table 1) promote actinobacterial growth because of their versatile metabolic capabilities compared to other bacteria and may also promote their sporulation. B-vitamins, known as growth factors for certain Actinobacteria, were also utilized (Figures 1, 2 and Table 1; Kurtböke, 2003). Antibiotics act as additional selectors favoring Actinobacteria as resourceful producers of the latter, which requires them to possess a large number of resistance genes in their antibiotic biosynthesis gene clusters (Peterson and Kaur, 2018). We succeeded to isolate 77 actinobacterial strains covering 10 genera, of which nine are considered rare. The genus *Micromonospora* is a prolific producer of diverse classes of antibiotic compounds, which have not yet reached a saturation point in discoveries yet (Boumehira et al., 2016). *Asanoa* is a rare genus within Micromonosporaceae and besides its frequent association to plants, specifically medicinal plants, few data are available on its antimicrobial potential (Qin et al., 2009; Niemhom et al., 2016). Several species from genus *Actinokineospora* are in recent focus of bioprospecting for their antimicrobial and antitrypanosomal activities (Abdelmohsen et al., 2014; Intra et al., 2016). More than a third of the described *Nocardia* species are opportunistic pathogens, however, they also produce industrially important antibiotic compounds and enzymes (Tanaka et al., 1997; Barka et al., 2016). *Leifsonia* has been isolated from Chinese and Indian medicinal plants, but information depth on natural products from this genus refer to the presence or absence of synthetase classes and antibiotic sensitivity patterns (Qiu et al., 2007; Passari et al., 2015). *Micrococcus* can have plant growth promoting abilities as endophyte, e.g., in ginseng (Vendan et al., 2010) and is known to produce pigments with antimicrobial activity and other antibiotic compounds (Palomo et al., 2013; Umadevi and Krishnaveni, 2013). *Kitasatospora* is recognized as versatile producer of secondary metabolites of highly diverse nature, such as anti-tumor agents, immune modulators, anti-virals, herbicidals, and anti-protozoan compounds (Takahashi, 2017).

Host plant species exert a strong selective effect on their rhizosphere and endophyte microbiome (Ofek-Lalzar et al., 2014; Kumar et al., 2017), whereby endophytes often originate in the rhizosphere soil (Compant et al., 2010; Hardoim et al., 2015). Therefore, sequence data from rhizosphere and endosphere of the host plant *L. nivale* subsp. *alpinum* were jointly analyzed using a phylogenetic approach (Figure 3) to explore the endophytic Actinobacteria community. Pooling plant individuals among the investigated tissues prevents conclusions on differences of Actinobacteria communities associated to each plant specimen, however, this exceeded the focus of this study. Actinobacteria genera were found in all investigated plant organs with decreasing diversity from roots (292 OTUs), rhizomes (213), leaves (29), and inflorescences (11) based on a cutoff at 97% identity (Figure 3B). Rhizosphere isolates were well-represented among endophyte OTUs (Figure 3A), however, limited resolution of endophyte OTUs due to short sequence length and differing error rates do not allow a numerical estimation of the overlap between endo- and rhizosphere Actinobacteria. Rhizosphere isolated exclusively occurred in clade C (Actinomycetales), but were absent from clades A (Rubrobacteridae and Thermoleophilales), B (Acidomicrobiales), and D (Unclassified) (Figure 3A). Rubrobacteridae and Acidomicrobiales are known for the formation of micro-colonies of less than 25 μm with extremely slow growth rates supporting an isolation bias against this clade (Davis et al., 2011). Thermoleophilales are typically extremophiles. Clades A and B contain members of six assigned genera: *Conexibacter*, *Solirubrobacter*, and *Thermoleophilum* (clade A), *Aciditerrimonas*, *Iamia*, and *Illumatobacter* (clade B) as well as a large proportion of unclassified OTUs (Figure 3 and Supplementary Figure 1). Members of clade A and B have been rarely isolated from plants, but do occur in metagenomic endophyte studies (Pereira et al., 2017; Wemheuer et al., 2018). This suggests a pervasive culture bias against members of both clades as a more likely scenario rather than a selective enrichment of members of clade A and B in the endosphere. A culture and/or study bias is also supported by the uneven distribution of OTUs based on genus-level taxonomic assignment with almost 90% of assigned genera housed in clade C and is supportive of the notion that large sections of the phylogenetic diversity of Actinobacteria remain yet to be described (Gao and Gupta, 2012).

On a higher taxonomic level, Actinobacteria family diversity based on metabarcoding data suggests two sub-groups within different plant tissues: highly diverse belowground communities in rhizomes and roots and less diverse communities in aboveground tissues dominated by different families of Actinobacteria (Figure 4). In order to confirm differences in taxonomic distribution among tissues and assess inter-individual variation in endophytic diversity, fully replicated metabarcoding designs will be required.

This study describes the isolation of Actinobacteria from the rhizosphere of an alpine medicinal plant, which will be explored for drug discovery by genome- and culture-based bioprospecting approaches in the future. Moreover, our results provide an initial view of taxonomic representation of Actinobacteria occurring endogenously in *L. nivale* subsp. *alpinum*. Few studies are available on Actinobacteria associated to alpine plants, but those available promote a selective effect of host plant species, especially within the same site (Sheng et al., 2010; Ciccazzo et al., 2014; Chang et al., 2018). Our data is preliminary in this respect and further studies including multiple sampling sites are required to address possible site effects of encountered microbiota. Generally, *Streptomyces*, *Micromonospora*, and *Nocardia* commonly occur as endophytes in plants (Qin et al., 2010) and are also part of rhizo- and endosphere assemblies in *L. nivale* subsp. *alpinum* in this study. *Artemisia annua* (Asteraceae), a related medicinal plant, shares the endophytic genera *Micrococcus*, *Nocardia*, and *Streptomyces* (Li et al., 2011; Figure 3). Taechowisan et al. (2003) isolated Actinobacterial endophytes from 36 medicinal plants and retrieved only four different genera including all of the above mentioned common genera plus *Microbispora*. In contrast, the genera *Asanoa*, *Aktinokineospora*, *Mycobacterium*, *Micrococcus*, *Leifsonia*, *Microbacterium*, and *Kitasatospora* are less commonly encountered. From a bioprospecting perspective, all Actinobacteria isolated in this study but *Streptomyces* can be considered as rare and promising candidates for further research (Kurtböke, 2003). Metabarcoding data highlight the presence of numerous yet undescribed endophytic Actinobacteria belonging to deep rooting clades A, B, and D, which were detected in roots and rhizomes of Edelweiss plants (Figure 3 and Supplementary Figure 1), but have rarely been isolated from plants before with the exception of *Solirubrobacter phytolaccaceae*, *S. ginsenosidimutans*, *Patulibacter brassicae*, and *P. ginsengiterrae* (An et al., 2011; Kim et al., 2012; Wei et al., 2014; Jin et al., 2016). Based on these insights, future investigations may specifically consider isolation of Actinobacteria from its belowground tissues, which bear large amounts of this uninterrogated diversity. Selective isolation efforts may be tailored to targeting specifically clades A, B, and D, which promise larger taxonomical depth than currently known and would greatly expand our knowledge on Actinobacterial diversity, potentially including many new producers of novel metabolites.

## Data Availability Statement

The datasets generated for this study can be found in the GenBank MH513505 – MH513581 (publication date July 15, 2019), https://github.com/JackyHess/Leontopodium_metagen, European Nucleotide Archive PRJEB32100 (ERP114738), alignments and phylogenies are made available at Dryad Digital Depository^6^.

## Author Contributions

MO, SZ, CW, TR, and JH contributed to the concept and design of this research. TR and JH conducted the bioinformatics analyses. MO, ML, and FG isolated Actinobacteria from the rhizosphere soil and genotyped them. MO, JH, and ML analyzed the phylogenetic relationship of isolates. MO, JH, and SZ drafted the manuscript. All authors critically revised it and agreed to all aspects of the work presented.

## Conflict of Interest

The authors declare that the research was conducted in the absence of any commercial or financial relationships that could be construed as a potential conflict of interest.

## Footnotes

1 https://www.dsmz.de/microorganisms/medium/pdf/DSMZ_Medium12.pdf

2 http://www.straininfo.net

3 https://doi.org/10.5061/dryad.mkkwh70vg

4 https://www.arb-silva.de

5 https://github.com/JackyHess/Leontopodium_metagen

6 https://doi.org/10.5061/dryad.mkkwh70vg
