# Supplementary figures and images for: Exploring Actinobacteria Associated With Rhizosphere and Endosphere of the Native Alpine Medicinal Plant Leontopodium nivale Subspecies alpinum
Source: Front Microbiol. 2019 Nov 8;10:2531. doi: 10.3389/fmicb.2019.02531 (PMC6857621; doi:10.3389/fmicb.2019.02531)

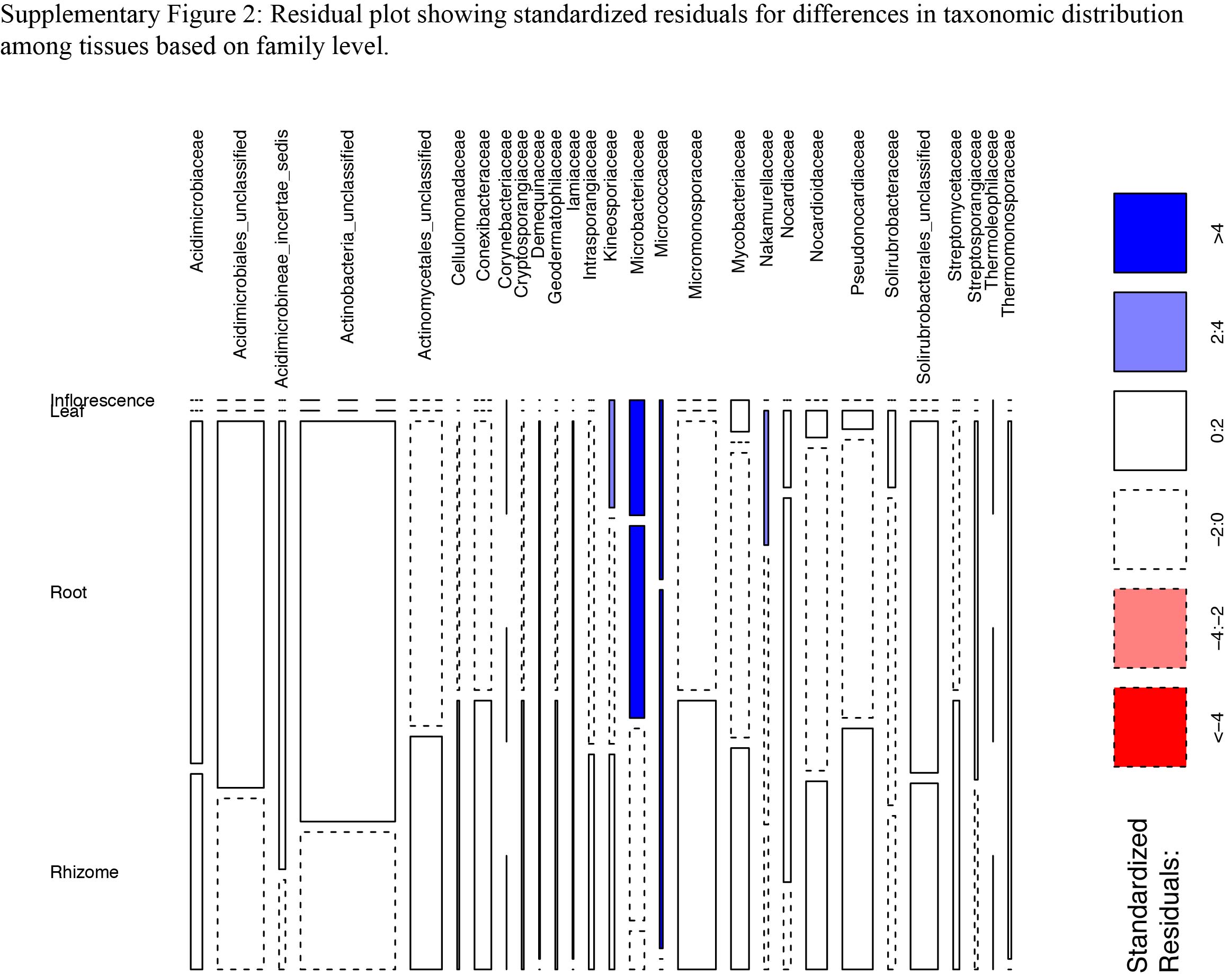

Supplement: Supplementary file 2 [file Image_2.JPEG]
